# Loss of Nocturnin increases neuronal viability in oxidative stress conditions

**DOI:** 10.3389/fnins.2025.1632251

**Published:** 2025-11-19

**Authors:** Anne E. Ojo, Crystal Olivas-Rasmussen, Samuel S. Pappas, Carla B. Green

**Affiliations:** 1 Department of Neuroscience, UT Southwestern Medical Center, Dallas, TX, United States; 2 O’Donnell Brain Institute, UT Southwestern Medical Center, Dallas, TX, United States; 3 Department of Neurology, UT Southwestern Medical Center, Dallas, TX, United States

**Keywords:** Nocturnin, oxidative stress, Parkinson’s, ROS, NADPH, glutathione, circadian

## Abstract

Oxidative stress, characterized by an imbalance between reactive oxygen species (ROS) and antioxidants, plays a critical role in neurodegenerative disorders like Parkinson’s Disease (PD) and is strongly associated with neuronal cell death. Nocturnin was identified as a NADP(H) phosphatase and key regulator of oxidative stress. NADPH serves as a crucial co-factor for enzymes which regenerate antioxidants, and downregulation of its levels increases sensitivity to oxidative stress mediated neurodegeneration. In this study, we examined how the loss of Nocturnin impacts redox homeostasis and neuronal survival in Cath.a-differentiated (CAD) cells and dopaminergic neurodegeneration in a mutant alpha-synuclein overexpression PD mouse model (DA_SYN53_). Here we demonstrate that loss of Nocturnin increases CAD cell viability by increasing total glutathione levels, boosting metabolites involved in antioxidant defense, and reducing oxidative damage. Additionally, Nocturnin deletion in DA_SYN53_ mice promotes midbrain dopaminergic neuron survival. These findings suggest that the loss of Nocturnin protects neurons from oxidative stress by increasing antioxidant defense, which rescues neurodegeneration of dopaminergic neurons.

## Introduction

The complexity of neurodegenerative disorders (NDDs) has made it difficult to understand disease development and develop therapeutics. Oxidative stress, a cellular state with a significant increase in reactive oxygen species (ROS) and a decrease in antioxidant levels, is an underlying component of disease progression implicated in multiple NDDs and contributes to the complexity of NDDs (Barnham et al., 2004; Kim et al., 2015). Cells regulate ROS and antioxidant production through reduction–oxidation (redox) homeostasis, which is fundamental for nearly all physiological processes. In redox homeostasis, when ROS levels are acutely and purposely increased, they act as signaling molecules to activate processes such as proliferation, inflammation, and autophagy. Overproduction or mistimed ROS accumulation can create a highly oxidizing environment and lead to mitochondrial dysfunction, autophagy inhibition, protein aggregation, and eventual cell death if not rectified (Sies et al., 2017). These pathophysiological phenotypes are particularly notable in Parkinson’s Disease (PD), the second most common NDD. In PD, dopaminergic neurons in the substantia nigra pars compacta (SNc) undergo significant neurodegeneration at early stages, followed by nearby ventral tegmental area (VTA), and progression to cortical regions at later stages of the disease (Damier et al., 1999; Poewe et al., 2017). Dopamine, while an essential neurotransmitter, plays a paradoxical role within dopaminergic neurons due to its metabolism, which can lead to the production of ROS and toxic byproducts. Dopamine oxidation generates adducts that exacerbate oxidative stress, which can promote the aggregation of alpha-synuclein—a key factor in the progression of some PD cases (Surmeier, 2018; Wise et al., 2022). However, while the dopaminergic characteristic of neurons affected in PD is a distinguishing feature compared to neurons in other NDDs, dopamine itself is not the sole factor underlying their vulnerability. Oxidative stress in PD is noteworthy as it is a prominent feature in both familial and sporadic cases of PD and is closely linked to additional pathogenic mechanisms seen in both types of PD, including mitochondrial dysfunction, iron accumulation, and neuroinflammation (Yang et al., 2025).

A key strategy for preventing oxidative stress is the use of antioxidants, which are central to regulating ROS levels. Glutathione is one of the most abundant and versatile antioxidant molecules used in the brain to mitigate cellular ROS and is significantly decreased in Parkinson’s patients (Aoyama, 2021; Sofic et al., 1992). Glutathione is synthesized through several pathways, but to rapidly combat ROS, reduced glutathione (GSH) is oxidized into glutathione disulfide (GSSG) by antioxidant enzymes. GSSG is regenerated back into its usable reduced form by the enzyme glutathione reductase (GR) using the reducing power from nicotinamide adenine dinucleotide phosphate (NADPH; Averill-Bates, 2023). Decreased activity of pathways responsible for the reduction or biosynthesis of NADP(H) has been shown to sensitize cells to oxidative stress and cell death (Balsa et al., 2020; Dunn et al., 2014; Ying, 2007). Interestingly, in an MPTP-induced Parkinsonian mouse model, NADPH intraperitoneal injections following MPTP administration reduced ROS and increased GSH levels (Zhou et al., 2018), suggesting that NADPH contributes to cellular health in PD.

The circadian protein Nocturnin is a NADP(H) phosphatase, that converts NADP(H) into NAD(H) (Abshire et al., 2020; Estrella et al., 2019; Laothamatas et al., 2020). The purpose of this phosphatase activity has not been fully elucidated, however, there are several reports that have associated Nocturnin in oxidative stress development. Overexpressing Nocturnin significantly decreases NADP(H) levels and increases susceptibility to oxidative stress conditions and cytotoxicity, while loss of Nocturnin was protective in HEK293 cells (Laothamatas et al., 2020). Additionally, Nocturnin expression is increased in dopamine neurons isolated from postmortem brains of PD patients (Simunovic et al., 2008) and increased in striatal spiny projection neurons following dopamine depletion and chronic L-DOPA treatment in models of dyskinesia (Charbonnier-Beaupel et al., 2015; Heiman et al., 2014). Studies in *Drosophila* further link Nocturnin and cellular dysfunction in PD. Loss of *pink1*, a gene associated with autosomal recessive early onset PD (Valente et al., 2004), in *Drosophila* cells causes excessive mitochondrial fusion (Yang et al., 2008), which is rescued by Nocturnin knockdown (Pogson et al., 2014). Collectively, these data suggest that Nocturnin modifies cellular dysfunction in PD, but its role in dopaminergic neuron degeneration is unknown.

To determine Nocturnin’s role in oxidative stress-mediated neurodegeneration, we investigated Nocturnin’s role in a catecholaminergic neuronal cell line under normal and induced oxidative stress conditions to examine how loss of Nocturnin affects redox homeostasis and neuronal viability. We discovered that loss of Nocturnin increases cell viability through increasing antioxidant levels and antioxidant related metabolites, which results in less ROS accumulation and oxidative damage. Additionally, we deleted Nocturnin in a mutant alpha-synuclein overexpression PD mouse model, which significantly increased dopaminergic neuron viability *in vivo*. Understanding how loss of Nocturnin reduces susceptibility to oxidative stress may provide insight into disease onset and progression for PD and other NDDs characterized by oxidative stress prior to cell death. Nocturnin’s role as a NADP(H) phosphatase may provide an alternative approach toward treatment of these disorders.

## Methods

### Cell culture

Cath.a-differentiated (CAD) cells were purchased from Sigma-Aldrich and maintained in Dulbecco’s modified Eagle’s medium (DMEM)/F12, 8% Fetal Bovine Serum (FBS), 1% penicillin/streptomycin (P/S) antibiotics at 37 °C and 5% CO_2_. Nocturnin KD CAD cells (shNoct) were generated by transducing lentivirus containing the pLKO.1 vector (Sigma-Aldrich) targeting the 3’UTR region of *Nocturnin* (target sequence: GCACTCCAGTTTGAGCTTGTT), for 48 h. Successfully transduced cells were selected with 5 mg/mL puromycin for 1 week and knockdown of Nocturnin was confirmed via western blot. For overexpression experiments, 250,000 cells in 6-well plates were transfected with pCMV-E193A-NOCT-mCherry using Lipofectamine 3000 for 24 h. The overexpression was confirmed by visualization of the mCherry signal using a Cytation 10 (Agilent BioTek) plate reader using the Texas Red fluorescence Ex/Em. CAD cells were plated at cell density as indicated in each individual experiment and allowed to reattach overnight. The LUNA-II cell counter (Logos biosystems) or a hemocytometer was used to count cell densities for cell plating.

### Animals

All animal work described in this manuscript has been approved and conducted under the oversight of the UT Southwestern Institutional Animal Care and Use Committee. Male and female mice used for the experiments were group housed in temperature- and humidity-controlled rooms on a 12 h light/dark cycle (lights on at 7a.m.) with *ad libitum* food and water. Double transgenic “DA_SYN53_” mice [DAT-PF-tTA; Tg(tetO-SNCA X A53T); Chen et al., 2015] were generously provided by William Dauer, MD. Mice were maintained without doxycycline treatment to enable alpha synuclein expression in this “tet-off” system. Nocturnin knockout DA_SYN53_ mice (DA_SYN53_; Nocturnin^−/−^) were derived from crossing DA_SYN53_ mice with whole body Nocturnin^−/−^ mice (Green et al., 2007).

### Cellular viability assay

WT and shNoct CAD cells were plated at 20,000 cells per well in a 96-well black plate/clear bottom. After 24 h, the cells were treated with 0-300 μM H_2_O_2_ and allowed to incubate for 4 h. After incubation, AquaBluer detection reagent (MultiTarget Pharmaceuticals) was conducted according to manufacturer’s instructions. Cells were washed two times with PBS, AquaBluer was diluted 1:100 in culture medium, and 100 μL added to each well. After 3 h of incubation with AquaBluer, the fluorescence signal (540ex/590em) was measured using a Biotek microplate reader (Agilent).

### Measurement of NAD+/NADH, NADP+/NADPH, and GSH/GSSG ratios

For the NADP+/NADPH kits (Promega), a total of 30,000 cells were plated in a 96-well plate and allowed to reattach overnight. The following day, the cells were treated and incubated with 123 μM H_2_O_2_ for 4 h. Cells were processed according to manufacturer’s instructions. Media was removed, PBS was added to each well, and cells were then lysed with base solution and 1% DTAB for 5 mins on a shaker. The lysed solution was divided into half and used for either NADP+ or NADPH measurements. According to manufacturer’s instructions, samples used for NADP+ were acid treated and heated while samples used for NADPH measurement were only heated. Acid in the NADP+ samples were neutralized with Trizma, while NADPH samples received HCL/Trizma. NADP+/NADPH-Glo Detection Reagent was added to each well, the plate was incubated at room temperature for 45 min, and luminescence was measured on Biotek plate reader (Agilent). For the GSH/GSSG ratios or individual GSH and GSSG measurements, 20,000 cells were plated per well in a 96-well plate and allowed to reattach overnight. Wells were plated for GSH and GSSG measurements separately. The following day, the cells were treated with 123 μM H_2_O_2_ and incubated as indicated in each individual experiment. Afterwards, the GSH/GSSG-Glo assay was performed according to manufacturer’s protocol (Promega). Media was removed from each well, and either total glutathione or oxidized glutathione Lysis Reagent was added. The plate was incubated at room temperature on a shaker and then Luciferian Generation Reagent was added to every well. Plate was shaken at room temperature for 5 min, then incubated for another 25 min at room temperature. Luciferin Detection Reagent was added to every well, and after a 15 min incubation at room temperature, luminescence was measured using the Biotek plate reader (Agilent).

### Measurement of ROS

WT and shNoct CAD cells were plated at 600,000 cells per well in a 6-well plate, and after 24 h, cells were treated with 123 μM H_2_O_2_ for 1 h. Cells were then stained with CellROX Green reagent (Thermo Fisher) according to manufacturer’s instructions. CellROX Green was diluted to a final concentration of 5 μM and incubated for 30 min at 37° in the incubator. Cells were washed with PBS three times and then collected in HBSS. An undyed control was also prepared for the flow analysis. Stained samples were analyzed on a FACSCalibur (BD Biosciences) equipped with a 488 nm laser for excitation of CellROX Green and a 530/30 BP filter for collection of fluorescence emission. Events/s was 2,500 cells/s and a minimum of 20,000 cells was collected. Data was analyzed using FlowJo 10.10.0 software (Beckton Dickinson).

### Western blots

Cells were collected, washed in 1X PBS, lysed, agitated in 1X RIPA buffer (50 mM Tris-pH8, 150 mM NaCl, 1% TritonX-100, 0.5% sodium deoxycholate, 1% SDS, and 1X cOmplete™, Mini Protease Inhibitor Cocktail) for 30 min at 4 °C, and then centrifuged at 15,000 x g for 15 min. Alternatively, for the collection of substantia nigra (SN) and VTA tissue, freshly dissected mouse brains were sliced using a vibratome. Two 600 μm slices, each containing the SN and VTA, were collected and both regions were carefully isolated from the rest of the brain slice. The tissue containing the SN and VTA was then rapidly flash-frozen in liquid nitrogen before they were homogenized in 1X RIPA buffer with a 25-gauge needle. The supernatant was transferred to a new tube and protein concentration was determined using a Pierce BCA Protein Assay kit (Fisher Scientific) according to manufacturer’s instructions. Protein samples were then diluted in 5X Laemmli loading buffer and heated at 95 °C for 5 mins. Samples were loaded into a 4–20% Mini-PROTEAN TGX precast protein gel (Bio-Rad) and run at 80 V constant for 2 h and then transferred to polyvinylidene fluoride membranes for 1.5 h at 100 V. Membranes were blocked with filtered 3% BSA/TBST for 1 h, followed by overnight incubation at 4 °C with one of the following primary antibodies diluted in 3% BSA/0.5% sodium azide: β3-Tubulin (Cell Signaling Technology), alpha synuclein-Syn204 (Cell Signaling Technology), Mouse alpha Synuclein Antibody (Cell Signaling Technology) or *α*-Actin (EMD Millipore) were diluted to 1:1000. In addition, we generated a rabbit Nocturnin primary antibody which was diluted to 1:2000. The following day, the membranes were washed four times in TBST for 10 min each and then incubated anti-rabbit HRP diluted to 1:2000 in 3% BSA/0.5% sodium azide. Blots were visualized with Clarity Max Western ECL (Bio-Rad) and Chemidoc MP Imaging System (Biorad). Blots were analyzed using Image Lab software.

### Metabolomics

WT and shNoct CAD cells were plated at 2–3 million cells per 10-cm plate 24 h before collection. One hour before collection, oxidatively stressed CAD cells were treated with 123 μM H_2_O_2_. At collections, cells were washed with ice-cold saline, then lysed with 80% acetonitrile in water, and then scraped into an Eppendorf tube. The cells went through three freeze–thaw cycles in liquid nitrogen, centrifuged at 20,160 x g for 15 min at 4 °C, and the supernatant was transferred into a new tube. Protein concentration was determined by the Pierce BCA kit (Fisher Scientific), and then 10 mg of protein was transferred to a new tube for each sample. The samples were centrifuged one final time at 20,160 x g for 15 min at 4 °C, and the final supernatant was transferred to a new tube and stored at −80 °C for less than 24 h before the supernatant was analyzed using liquid chromatography–tandem mass spectrometry (LC–MS/MS). Data acquisition for metabolomics was completed as reported previously (Aurora et al., 2022; Tasdogan et al., 2020). Metabolites were quantified by CompoundDiscoverer 3.3 SP3 (Thermo Scientific) searching against an in-house metabolite library. Metabolites from experimental samples were identified by matching four separate metrics of previously characterized metabolites in our library: monoisotopic precursor ion mass, natural isotopic distribution, MS2 spectra, and retention time by our HILIC chromatography method. Metabolites that scored above 50% using these four metrics were manually reviewed for accuracy to generate a final list of metabolites. After export, peak areas for metabolites were analyzed by Metaboanalyst for statistical significance and pathway analysis. Heatmaps and pathway enrichment was generated using Metaboanalyst.

### TBARS assay

WT and shNoct CAD cells were plated at 10 million cells in a 10-cm plate 24 h before collection for the TBARS Assay kit (Cayman Chemical). Plates were incubated with 123 μM H_2_O_2_, and were treated 1 and 4 h before cells were collected. Cells were washed with 1X PBS and then collected with 1X PBS. Cells were centrifuged down at 1,500 x g for 5 min and then PBS was removed. The cells were then homogenized in 1X RIPA buffer and then allowed to agitate for 15 min at 4 °C. The homogenized samples were processed according to manufacturer’s instructions using the colorimetric standard preparation. Absorbance was read at 532 nm on the Biotek plate reader (Agilent). Protein concentration of the samples was measured using the Pierce BCA Protein Assay kit (Fisher Scientific).

### Immunohistochemistry

Six-month-old male and female mice were anesthetized with isoflurane and transcardially perfused with cold 1X PBS followed by 4% PFA/PBS (Santa Cruz Biotechnology). Mouse brains were postfixed in 4% PFA/PBS overnight at 4 °C followed by 10% sucrose/PBS. The brains were moved to increasing sucrose/PBS concentrations up to 30% sucrose/PBS. 40 μm tissue sections were sliced on a microtome and collected in a series of three in PBS/0.1% sodium azide. Tissue sections were washed in 1X TBS, incubated in 0.05 M sodium citrate, blocked in 0.1 M Glycine, and blocked in concentration blocking solution (1X TBS, 0.4% Triton X-100, 1% H_2_O_2_, and 10% NGS). Following blocking, sections were washed and incubated in the following primary antibodies diluted in TTG (1X TBS, 0.4% Triton X-100, and 2% NGS) for 24 h: chicken anti-tyrosine hydroxylase (Aves Labs; 1:2000) and rabbit anti-NeuN (Cell Signaling Technologies; 1:500). Slices used for stereology were incubated in goat anti-chicken Alexa Fluor 488 secondary antibody (Jackson ImmunoResearch; 1:250) or donkey anti-rabbit Alexa Fluor 647 secondary antibody (Invitrogen; 1:800) for 1 h. Tissue sections were mounted with Vibrance Antifade Mounting Media (Vector Laboratories) and allowed to dry for at least 48 h.

### Stereology

TH positive cells from immunohistochemistry sections were quantified blinded with unbiased stereological cell counting using the StereoInvestigator Optical Fractionator Probe (MBF Bioscience). TH- and NeuN-stained sections observed with an AxioImager M2 (Zeiss) were outlined using a 10x objection (*n* = 4–6 sections observed for each brain). Cells were counted using a 63x oil immersion objective with a 2 μm guard zones, 180 × 180 μm grid, and 60 × 60 μm counting frame. The top of the nucleus was used as the point of reference.

### Experimental design and statistical analysis

Tests of normality and homogeneity of variance were checked, and the appropriate tests were applied to analyze differences between genetic and treatment groups. The data is presented as the mean ± SEM (Supplementary data sheet 1), and differences were analyzed using an independent *t* test, Welch’s t-test, Mann–Whitney, one-way ANOVA followed by a Tukey *post-hoc* test, or a two-way ANOVA followed by Sidak *post-hoc* test as indicated. Statistically significant results were defined as follows: *p < 0.05; **p < 0.05; ***p < 0.001, and ****p < 0.0001.

## Results

### Loss of Nocturnin increases cell viability in CAD cells

**Figure 1:**
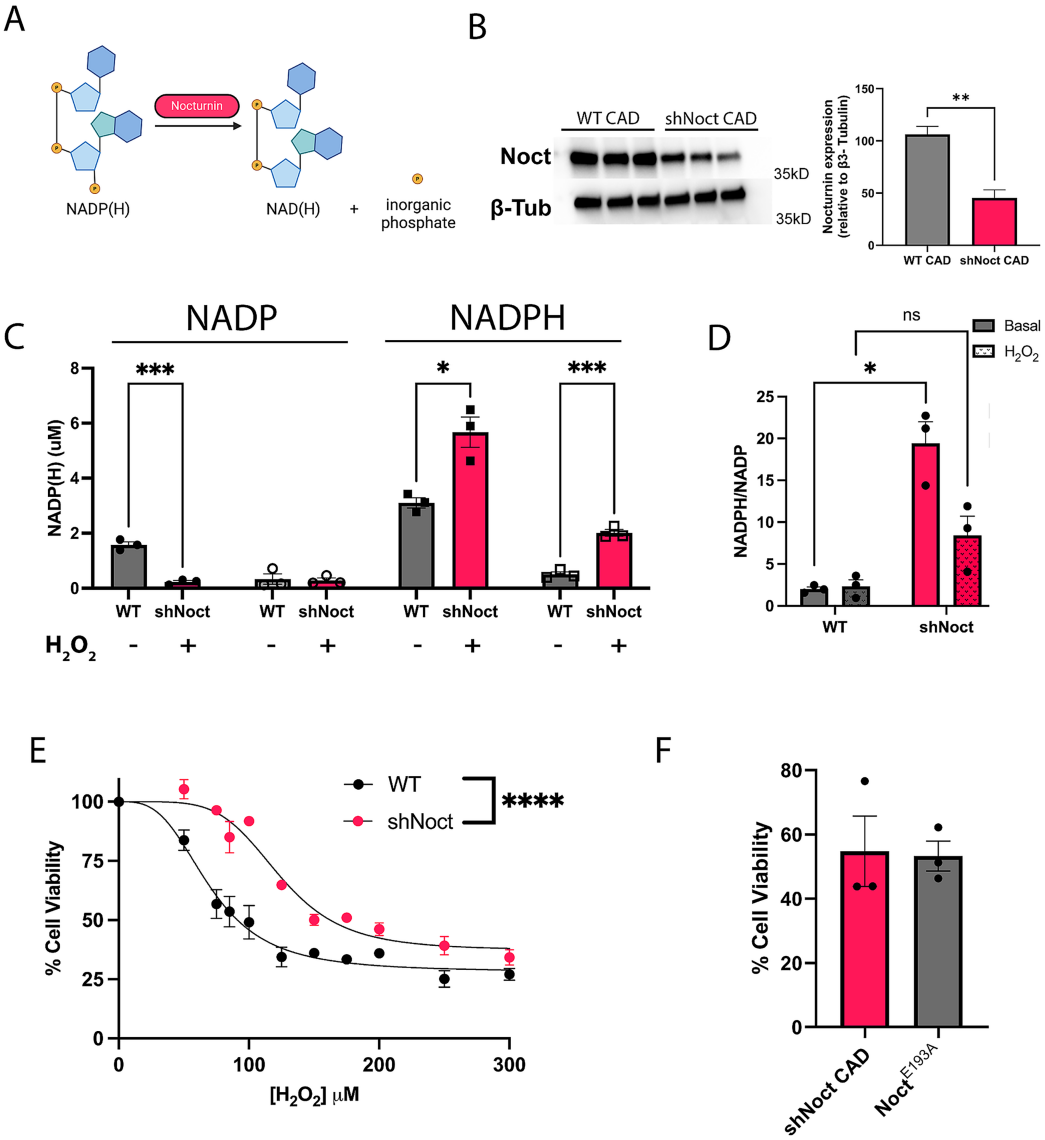
Nocturnin knockdown increases NADPH levels and CAD cell survival during oxidative stress conditions. **(A)** Outline of Nocturnin’s NADP(H) phosphatase activity. Created with BioRender.com. **(B)** Western blot and quantification of Nocturnin expression in WTand shNoct CAD cells. β3-Tubulin was used as a loading control. Unpaired t-test; *N* = 3. **(C,D)** NADP(H) measurements **(C)** and NADPH/NADP+ ratio **(D)** in WT and shNoct CAD cells in basal and H_2_O_2_ oxidative stress induced conditions at 123 μM for 4 h. Unpaired t-test for each metabolite/treatment group; *N* = 3 **(C)**. Welch’s *t*-test; *N* = 3 **(D)**. **(E)** Cell viability of WT and shNoct CAD cells incubated with increasing H_2_O_2_ concentrations for 4 h and measured with AquaBluer. Non-linear fit; *n* = 4. **(F)** Cell viability of shNoct and E193A Noct CAD cells (in shNoct background) treated with 123 μM H_2_O_2_ for 4 h. Unpaired t-test; *N* = 3. Statistically significant results were defined as follows for the figure: *p < 0.05; **p < 0.05; ***p < 0.001, and ****p < 0.0001.

Nocturnin functions as an NADP(H) phosphatase, removing the phosphate from both NADPH and NADP^+^ (Abshire et al., 2020; Estrella et al., 2019; Laothamatas et al., 2020; Figure 1A). However, the extent to which this activity affects neuronal cellular health and viability during stress is not known. To test if Nocturnin has a role in cellular response to stress in neuronal cell types, we created a stable Nocturnin knockdown (shNoct) cell line using Cath.-a-differentiated (CAD) cells (Figure 1B). Consistent with previous studies in non-neuronal cell lines (Laothamatas et al., 2020), NADPH levels are increased in the shNoct CAD cells compared to WT CAD cells in basal conditions (Figure 1C). When oxidative stress conditions are induced with hydrogen peroxide (H_2_O_2_), NADPH levels are decreased in both WT and shNoct CAD cells, but shNoct CAD cells maintain significantly higher levels than WT CAD cells (Figure 1C). Unexpectedly, basal NADP^+^ levels are significantly higher in WT CAD cells compared to the shNoct CAD cells (Figure 1C). However, in stressed conditions, NADP^+^ levels are similar in both WT and shNoct CAD cells. The NADPH/NADP^+^ ratio in shNoct CAD cells are significantly higher under basal conditions compared to WT CAD cells. A trend toward increased NADPH/NADP^+^ ratio was also observed under stressed conditions, but did not reach statistical significance (Figure 1D). We then examined the sensitivity of WT and shNoct CAD cells to oxidative stress by measuring cell viability as a function of increasing H_2_O_2_. These data show decreased levels of Nocturnin provide a protective effect against H_2_O_2_- induced cell death as the shNoct CAD cells have a curve shift significantly to the right, indicating less susceptibility to cytotoxicity and ROS-induced cell death (Figure 1E). The effective concentration for 50% loss of cell viability (EC_50_) in WT and shNOCT CAD cells shifted from 70 μM to 123 μM, respectively. This recapitulates reported data in HEK293 cells which showed NOCT KO resulted in protection against H_2_O_2_-induced cell death (Laothamatas et al., 2020). To determine whether this change in cell viability was indeed due to the phosphatase activity of NOCT, we over-expressed E193A NOCT,a mutation which disrupts the coordination of the active-site magnesium and renders the enzyme inactive. The cell viability of NOCT E193A overexpressing cells is identical to that of shNOCT CAD cells (Figure 1F). This indicates that the increase in cell viability conferred by knock-down of NOCT expression is due to NOCT’s activity. Therefore, loss of Nocturnin increases NADP(H) levels and survival in CAD cells subjected to oxidative stress.

### Lower Nocturnin levels increase glutathione levels and decrease ROS

**Figure 2:**
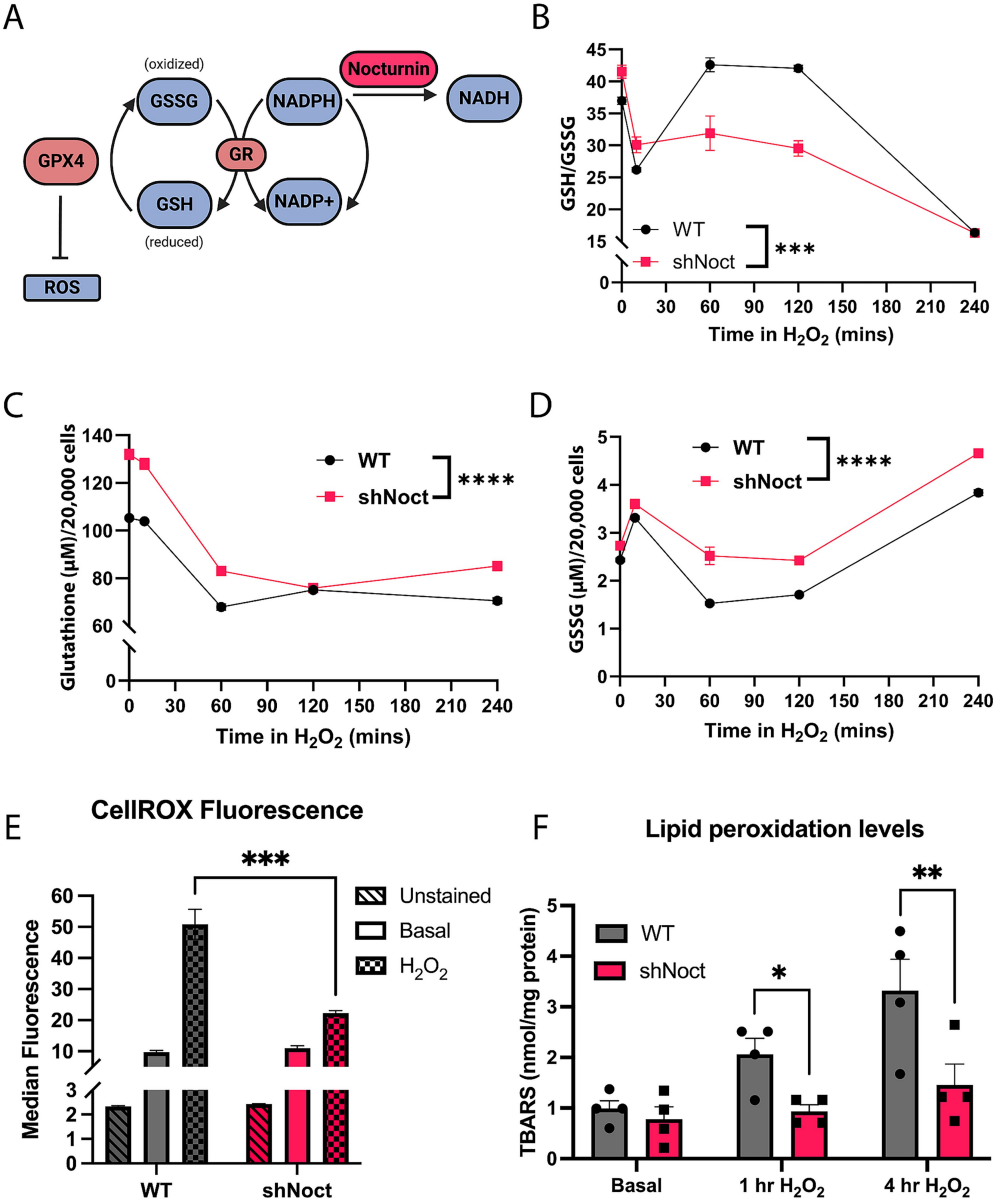
Total glutathione levels are increased while oxidative damage is decreased in shNoct CAD cells. **(A)** Outline of glutathione recycling and Nocturnin’s putative position in the pathway as an NADP(H) phosphatase. Created in BioRender.com. **(B–D)** Over a four-hour time course, WT and shNoct CAD cells were treated with 123 μM H_2_O_2_, and GSH/GSSG ratio **(B)**, total glutathione **(C)**, and GSSG **(D)** were measured. All experimental replicates and time-course data were conducted within a single day. Additionally, biological replicates (data not shown) were performed independently and yielded similar results, confirming the consistency of the observed effects; *N* = 6, Two-way ANOVA, Sidak. **(E)** Measurement of ROS levels in WT and shNoct CAD cells treated with 123 μM H_2_O_2_ for 1 h using CellROX Green. Unpaired t-test; *N* = 3. **(F)** Lipid peroxidation levels using the TBARS Assay kit, measured in WT and shNoct CAD cells in basal and stressed conditions (123 μM H_2_O_2_) for 1 or 4 h. Unpaired t-test for each treatment group; *N* = 4. Statistically significant results were defined as follows for the figure: *p < 0.05; **p < 0.05; ***p < 0.001, and ****p < 0.0001.

Cell survival under oxidative stress conditions largely depends on the cell’s ability to maintain redox homeostasis (Sies, 2019). Since NADPH is required for the regeneration of the functionally active GSH, we hypothesized shNoct CAD cells have improved survival during oxidative stress condition by increasing GSH levels, due to increased levels of NADPH (Figure 2A). Indeed, at basal conditions, GSH/GSSG ratios were significantly increased in shNoct CAD cells compared to WT CAD cells (Figure 2B). However, examination of the GSH/GSSG ratio over time following 1 h of H_2_O_2_ incubation results in WT CAD cells having significantly higher GSH/GSSG ratios, which we were not expecting to observe. We sought to better understand the contribution of the two redox forms of glutathione to the ratios, so we assessed total glutathione and GSSG levels separately. In WT and shNoct CAD cells, total glutathione levels decrease in the first hour of H_2_O_2_ incubation and then equilibrate, but shNoct CAD cells maintain significantly higher total glutathione levels compared to WT CAD cells (Figure 2C). GSSG levels are also significantly higher in shNoct CAD cells, with the largest difference at 1 h of H_2_O_2_ incubation (Figure 2D). Due to the increase in total glutathione levels, we tested if ROS levels were mitigated in shNoct CAD cells undergoing oxidative stress. We used CellROX Green dye which fluoresces upon oxidation and correlates with ROS levels in cells. There was no difference in ROS levels at basal conditions between WT and shNoct CAD cells; however, after 1 h of H_2_O_2_ treatment, shNoct CAD cells exhibit significantly less ROS accumulation compared to WT CAD cells (Figure 2E).

Due to the relative lower levels of ROS accumulation in shNoct CAD cells after H_2_O_2_ incubation, we investigated whether shNoct CAD cells had less oxidative damage compared to WT CAD cells. Lipid peroxidation is a common marker to identify oxidative damage in NDDs as the brain is particularly sensitive to lipid peroxidation, and glutathione is used by antioxidant enzymes such as GPX4 to combat increased lipid peroxidation (Fu et al., 2022; Peña-Bautista et al., 2019). We hypothesized that shNoct CAD cells would have less accumulation of the lipid peroxidation marker thiobarbituric acid reactive substances (TBARS) due to the increase in total glutathione levels. TBARS are compounds formed as a byproduct of lipid peroxidation. The TBARS assay utilizes the reaction between TBARS and thiobarbituric acid (TBA), which results in conjugates (TBA-TBARS) that can be measured fluorometrically. Therefore, the TBARS assay is a quantification of lipid peroxidation damage caused by ROS in the cell (Aguilar Diaz De Leon and Borges, 2020). We observed that WT and shNoct CAD cells at basal conditions had no difference in lipid peroxidation levels, which recapitulated the no difference in ROS levels we obtained. At 1 h of H_2_O_2_ treatment, there was a significant decrease in TBARS levels in the shNoct CAD cells compared to WT (Figure 2F). This significance was stronger by 4 h of H_2_O_2_ treatment, showing a significant decrease in lipid peroxidation in the shNoct CAD cells (Figure 2F). Overall, while GSH/GSSG ratios are only significantly increased in the shNoct CAD cells early in oxidative stress conditions, we observed that there is an overall increase in total glutathione levels, lower ROS accumulation, and decreased lipid peroxidation that contribute to the cellular survival in the shNoct CAD cells.

**Figure 3:**
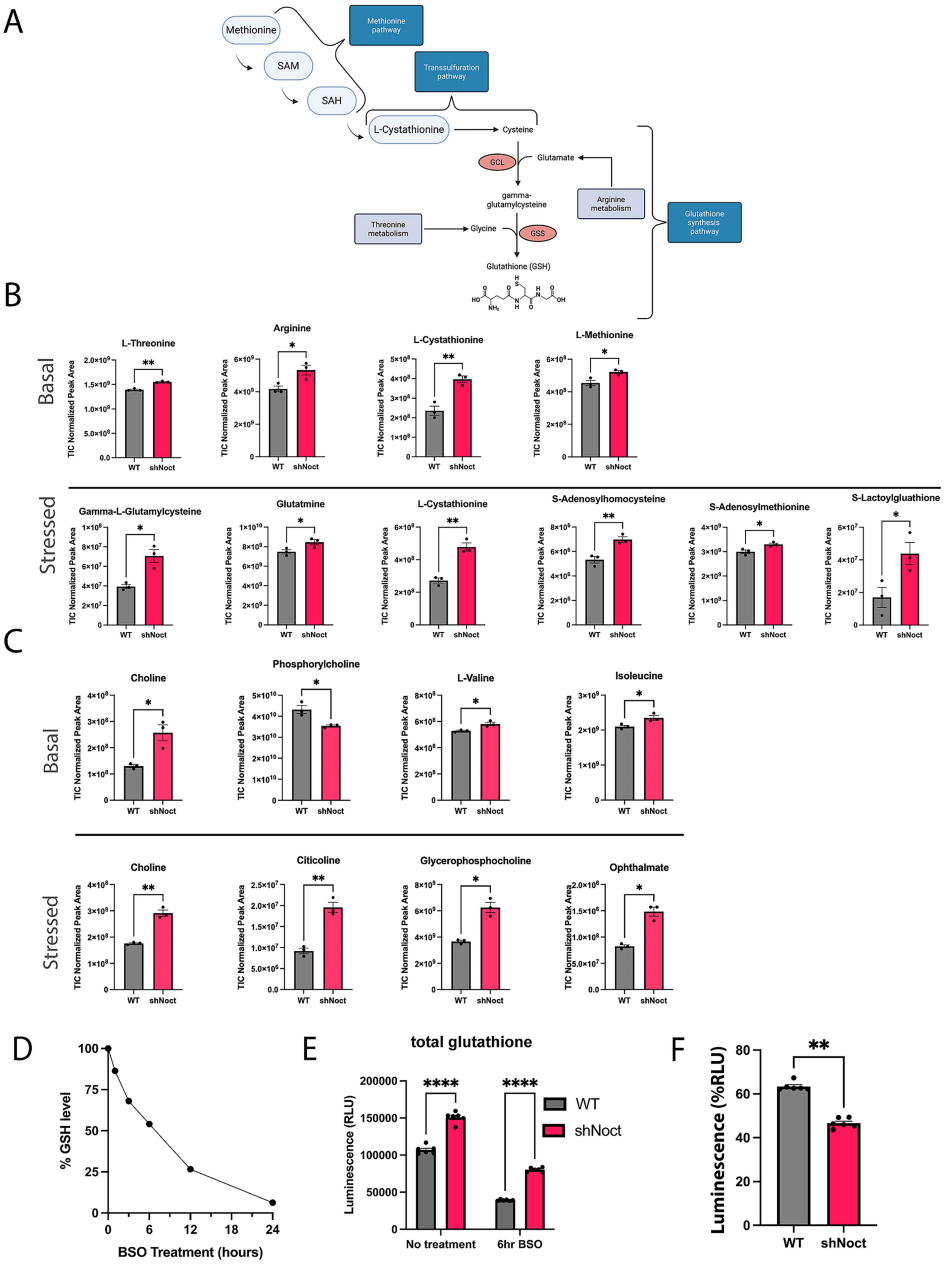
Metabolomic analysis shows loss of Nocturnin increases alternative glutathione synthesis metabolites. **(A)** Schematic of glutathione synthesis pathway, highlighting the points at which the identified metabolites from **(A)** are involved in the glutathione synthesis pathway. Created in BioRender.com. **(B)** Metabolites involved in the glutathione synthesis pathway identified from the metabolomics screen as significantly increased in shNoct CAD cells compared to WT CAD cells at basal and stressed conditions. **(C)** Metabolites involved in oxidative stress responses identified from the metabolomics screen as significantly increased in shNoct CAD cells compared to WT cells at basal and stressed conditions. **(D)** WT CAD cells treated with 1 mM BSO and total glutathione measured over a 24-h period. **(E)** Total glutathione measurements from WT and shNoct CAD cells that were treated with 1 mM BSO for 6 h Unpaired t-test; *N* = 6. **(F)** Percent of glutathione remaining calculated from total glutathione measurements via luminescence after 6-h BSO treatment in WT and shNoct CAD cells. Mann–Whitney; *N* = 6. Statistically significant results were defined as follows for the figure: *p < 0.05; **p < 0.05; ***p < 0.001, and ****p < 0.0001.

While we expected the GSH/GSSG ratios to increase in response to a loss of Nocturnin due to increased NADPH levels, we did not expect a significant increase in the total glutathione pool. We hypothesized that loss of Nocturnin is increasing total glutathione levels through adaptions in the glutathione synthesis pathway (Figure 3A). Therefore, we performed an untargeted metabolomics analysis to determine changes to metabolites and amino acids involved in the glutathione synthesis pathway. WT and shNoct CAD cells were treated with H_2_O_2_ for 1 h and collected along with a basal control for the metabolomics analysis (Supplementary data sheet 2). We observed an increase in the pathways that supply the building blocks for glutathione synthesis, and metabolic pathways identified to aid in oxidative stress response pathways (Figures 3A–C). The main amino acids involved in GSH synthesis are glutamate, glycine, and cysteine (Averill-Bates, 2023). At basal conditions, we observe a significant increase in arginine which can be converted into glutamate to support glutathione synthesis (Liang et al., 2018), and L-threonine which can be converted into glycine (Sahoo et al., 2021). In addition to finding metabolic sources of glycine and glutamate, we found metabolites involved in cysteine production through the transsulfuration pathway. The transsulfuration pathway provides synthesis of cysteine for glutathione synthesis (Vitvitsky et al., 2006). The methionine cycle is the precursor to the transsulfuration pathway and provides homocysteine, which is then used to initiate the transsulfuration pathway to produce L-cystathionine. Key metabolites in both the methionine and transsulfuration pathways were significantly increased at basal conditions (Figures 3A,B). Overall, this data corresponds to a significant increase in the intermediate metabolite gamma-glutamylcysteine at stressed conditions, which is also increased 1.3-fold at basal conditions in shNoct CAD cells (Supplementary data sheet 2). Therefore, the observed increase in total glutathione levels driven by the loss of Nocturnin is due to increased availability of amino acids upstream of glutathione synthesis.

To determine if WT and shNoct neurons used their glutathione stores at similar rates, we targeted the canonical glutathione synthesis pathway by treating WT and shNoct CAD cells with L-buthionine-(S, R)-sulfoximine (BSO) to inhibit glutamate-cysteine ligase (GCL; Griffith and Meister, 1979), the rate limiting enzyme in this pathway, and measured total glutathione levels. To determine the optimal incubation time to observe a significant loss of total glutathione in these cells, we first treated WT CAD cells with 1 mM of BSO over 24 h and measured total glutathione (Figure 3D). Based on this initial experiment, we chose a treatment of 1 mM BSO for 6 h, which caused a 50% reduction in steadystate glutathione levels and used this condition to treat WT and shNoct CAD cells. After BSO incubation, both WT and shNoct CAD cells have a significant decrease in total glutathione levels as expected, but total glutathione levels were still significantly elevated in the shNoct CAD cells compared to WT CAD cells (Figure 3E). The percent reduction in total glutathione levels after inhibition with BSO is significantly lower in shNoct CAD cells compared to WT CAD cells. This supports the hypothesis that lower Nocturnin levels contribute to higher total glutathione stores by using less glutathione, which suggests they are undergoing less oxidative stress (Figure 3F).

### Deletion of Nocturnin rescues dopamine neurons in a PD mouse model

**Figure 4:**
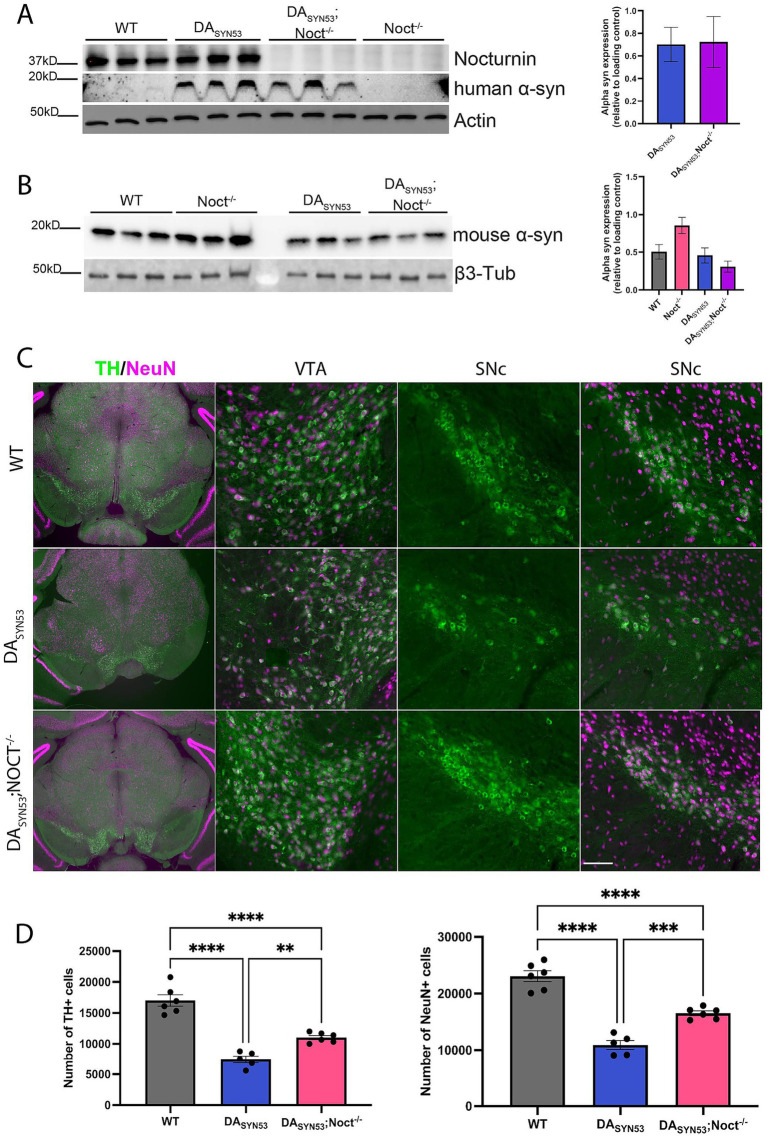
Loss of Nocturnin in DA_SYN53_ mice increases dopaminergic neuronal viability. **(A,B)** Western blot of Nocturnin **(A)**, human alpha-synuclein **(B)**, and mouse alpha-synuclein protein expression, with actin or beta3-tubulin used as a loading control from SN and VTA isolated from mouse brain tissue dissected from six-month-old WT, DA_SYN53_, and DA_SYN53_; Noct^−/−^ mice. Quantification of mouse alpha-synuclein is located to the right of the representative figures. One-way ANOVA, Tukey, *N* = 3/genotype. **(C)** Representative images of TH + (green) and NeuN+ (magenta) neuron populations from mouse brain sections used for stereology. First column is a 10x view of the tissue section, second column is a 20x view of the VTA, third column is a 20x view of only the TH + neurons of the SNc, and the fourth column is the same 20x view of the SNc with the NeuN+ population overlayed. Scale bar for first column: 500 mm; scale bar for second-fourth columns: 100 mm. **(D)** Unbiased stereological quantification of dopaminergic neuron numbers from mouse brain sections. One-way ANOVA, Tukey, *N* = 5–6 (4–6 sections per mouse). Statistically significant results were defined as follows for the figure: *p < 0.05; **p < 0.05; ***p < 0.001, and ****p < 0.0001.

Our CAD cell data shows improved cell survival and increased resistance to oxidative stress with Nocturnin knockdown, suggesting that reduction of Nocturnin levels could be a viable method to combat oxidative stress in NDDs such as PD. We tested whether the deletion of Nocturnin rescues neurodegeneration *in vivo* in a double transgenic PD mouse model overexpressing human mutant alpha-synuclein A53T selectively in dopaminergic neurons (DA_SYN53_). In this model, expression of tetO-alpha-synuclein A53T (tetO-SNCA^A53T^) is driven by the endogenous DAT promoter and an inserted tetO-tTA positive feedback (“PF”) gene expression amplification cassette, causing robust overexpression in the absence of doxycycline (Chen et al., 2015). While DA_SYN53_ mice do not exhibit significant motor behavioral phenotypes, DA_SYN53_ mice have a significant reduction of dopaminergic neurons at 6 months of age compared to WT mice, providing a quantitative histopathological endpoint (Chen et al., 2015). We hypothesized that deletion of Nocturnin in the DA_SYN53_; Noct^−/−^ mouse line would prevent or slow dopaminergic neuron loss in DA_SYN53_ mice. First, we collected SN and VTA tissue from six-month old WT, Noct^−/−^, DA_SYN53_, and DA_SYN53_; Noct^−/−^ mice to confirm Nocturnin expression in the WT and knockout of Nocturnin expression in the NOCT^−/−^. As expected_,_ Nocturnin expression was maintained in both WT and DA_SYN53_ mice, and was absent from DA_SYN53_; Noct^−/−^ mice (Figure 4A). Additionally, we tested for overexpression of human alpha-synuclein and endogenous mouse alpha-synuclein expression in the midbrain of these mice. Overexpression of human alpha-synuclein^A53T^ was confirmed in both DA_SYN53_ and DA_SYN53_; Noct^−/−^ mice (Figure 4A), while we observed a trend toward increased levels of endogenous mouse *α*-synuclein in the NOCT^−/−^but not in the DA_SYN53_ mice (Figure 4B).

To assess if DA_SYN53_; Noct^−/−^ mice had an increased survival of dopaminergic neurons, we used unbiased stereological cell counting to determine the number of tyrosine hydroxylase positive (TH+) dopaminergic neurons in the SNc and VTA. Consistent with previous reports, DA_SYN53_ mice at 6 months of age have significantly fewer TH + neurons compared to WT mice. DA_SYN53_; Noct^−/−^ mice have significantly more TH + neurons compared to DA_SYN53_, indicating a rescue of TH + cell loss (Figures 4C,D). To determine if the observed rescue of TH + neurons was attributable to a change in number of neurons rather than a change in TH enzyme expression, we labeled all neurons with NeuN antibodies and quantified NeuN+ neurons unbiased stereological cell counting. As expected, DA_SYN53_ mice have significantly fewer NeuN+ neurons compared to WT, which corresponds with the decrease in TH + staining (Figures 4C,D). DA_SYN53_; Noct^−/−^ have significantly more NeuN+ neurons compared to DA_SYN53_ mice, but were not fully restored to wild-type neuronal cell numbers (Figures 4C,D). Therefore, deletion of Nocturnin significantly mitigates cell death, increasing dopaminergic neuron survival in the DA_SYN53_ PD mouse model.

## Discussion

Oxidative stress, the imbalance of ROS and antioxidants, in NDDs is well established in the literature as both a consequence and promoter of disease progression. While production of ROS activates biological pathways for cellular homeostasis, a prolonged and pronounced presence of ROS exerts pathophysiological deleterious effects in the cell (Andersen, 2004; Barnham et al., 2004; Liu et al., 2017; Sies et al., 2024). The brain is more susceptible to the consequences of oxidative stress due to increased oxygen consumption, higher energy demand, and relatively reduced ability for cellular regeneration (Cobley et al., 2018; Mink et al., 1981). Dopaminergic neurons *in vivo* are particularly vulnerable, as both the metabolism and auto-oxidation of dopamine itself can augment ROS production (Panneton et al., 2010; Wise et al., 2022), contributing to oxidative stress mediated-neurodegeneration (Zuurbier et al., 2024). In this study, we demonstrate that decreasing the levels of the NADP(H) phosphatase, Nocturnin, mitigates oxidative stress and enhances dopaminergic neuron survival *in vitro* and *in vivo*. While Nocturnin is not a newly discovered protein (Green and Besharse, 1996), Nocturnin’s function as an NADP(H) phosphatase is more recent (Abshire et al., 2020; Estrella et al., 2019; Laothamatas et al., 2020). Consequently, Nocturnin’s role in redox homeostasis pathways and viability in neurons has not been studied.

Recent work has identified Nocturnin as a NADP(H) phosphatase that modulates oxidative stress development in HEK293 cells (Laothamatas et al., 2020). NADPH plays a significant role in maintaining redox homeostasis and cellular health. For example, NADPH oxidases that target NADPH produce significant levels of ROS for signal transduction, but can also promote neurodegeneration (Belarbi et al., 2017; Choi et al., 2012; Wu et al., 2003). Alternatively, NADPH is necessary for several NADPH-dependent reactions that regenerate antioxidants needed to maintain cellular health, and increasing NADPH has shown to be beneficial *in vitro* and *in vivo* (Miller et al., 2018; Zhou et al., 2018). In this study, we utilized Cath.a-differentiated (CAD) cells to investigate if loss of Nocturnin could affect neuronal cell survival. CAD cells are a CNS catecholaminergic cell line with neuronal and dopaminergic properties (Pasuit et al., 2004; Qi et al., 1997) that are used *in vitro* for their similarity to dopaminergic neurons and have robust endogenous expression of Nocturnin. Knockdown of Nocturnin expression in CAD cells results in a significant increase in NADPH compared to WT cells, which is also observed in H_2_O_2_-induced oxidative stress conditions. NADP^+^ is also a substrate of Nocturnin, but to a lesser extent (Estrella et al., 2019; Laothamatas et al., 2020), which supports why NADP^+^ levels are not increased in shNoct CAD cells compared to WT CAD cells (Figure 1C). However, upon treatment with H_2_O_2_, NADP^+^ levels do not increase in response to a decrease in NADPH levels in either cell line. The ratio of NADPH/NADP^+^ varies between cell types (Hedeskov et al., 1987; Zhang et al., 2015), but in WT CAD cells we observe the ratio to be closer to ~2 and is significantly increased with loss of Nocturnin (Figure 1D). Therefore, even under a shNoct background, the CAD cells maintain lower NADP^+^ levels during stress conditions. An additional hypothesis focuses on NADP^+^ involvement in several metabolic pathways, and therefore, can be shuttled to alternative pathways or transformed into new metabolites. For example, NADP^+^ can be converted into nicotinic acid adenine dinucleotide phosphate (NAADP) or cyclic ADPribose 2-phosphate (cADPRP), which regulate intracellular calcium release, which itself contributes to redox homeostasis via both production and detoxification of ROS (Agledal et al., 2010; Görlach et al., 2015). Therefore, NADP^+^ may be assisting in alternative pathways activated during oxidative stress development in CAD cells. This decrease in NADP^+^ is also reflected in our data as the ratio of NADPH/NADP^+^ is significantly increased following Nocturnin knockdown. In shNoct CAD cells, we aimed to investigate how the increase in NADPH levels affects the cell’s response to oxidative stress conditions. Here we show that the knockdown of Nocturnin increases CAD cell survival in oxidative stress conditions (Figure 1E). In addition, at basal conditions, GSH/GSSG is increased in shNoct CAD cells compared to WT CAD cells (Figure 2B). Additionally, shNoct CAD cells have elevated levels of total glutathione, in particular, and GSSG, compared to WT CAD cells after 1 h of H_2_O_2_ incubation. GSH/GSSG ratios were elevated in the shNOCT CAD cells (Figure 2B), therefore we assessed if this overall increase was significant enough to affect ROS levels and oxidative damage, a major downstream target of the glutathione antioxidant system. The inhibition of ROS and lipid peroxidation accumulation in the shNoct CAD cells (Figures 2E,F) suggests that the shNoct CAD cell survival is benefiting overall from the availability of total glutathione in the CAD cells. The observed changes in glutathione metabolism and oxidative stress resistance raise the possibility that Nocturnin deficiency may influence broader antioxidant response pathways. In particular, regulators such as NRF2 and related stress-responsive factors could be involved and merit further consideration in the context of this phenotype (Kaur and Aran, 2025). Overall, the discovery of Nocturnin’s role in the glutathione-dependent response pathways offers valuable insight into glutathione regulation, which is critical for understanding and potentially treating diseases with glutathione depletion.

A decline in GSH is linked to Parkinson’s disease advancement (Mischley et al., 2016). In this study, we show that loss of Nocturnin in CAD cells increases total glutathione pool (Figure 2C). Under both basal (vehicle only) and stressed conditions, we observed an increase in metabolites that support glutathione synthesis through the canonical and the alternative transsulfuration glutathione synthesis pathway (Figures 3A,B; Supplementary data sheet 2). In addition, we saw an increase in metabolites known to be involved in cellular stress responses such as the branch-chain amino acids (BCAAs) valine and isoleucine, the choline metabolism pathway, and the molecule ophthalmate (Figure 3C). For example, the branched-chain amino acids (BCAAs) valine, leucine, and isoleucine metabolic pathway are known to protect against oxidative damage (Liang et al., 2018; Sharma et al., 2023). Two of the metabolites in the BCAAs, valine and isoleucine, were significantly increased at basal conditions. Choline and derivatives of choline were also significantly increased at basal and stressed conditions. Choline has been shown to decrease oxidative stress, improve memory and motor functions in Alzheimer mouse models (Dave et al., 2023; Mehta et al., 2009). Additionally, the molecule ophthalmate, a derivative of glutathione, was significantly increased in stressed conditions. The role of ophthalmate is still under discussion, but several papers have suggested ophthalmate to be a positive and negative regulator of glutathione and potentially has the ability to mimic some oxidative response functions as glutathione (Schomakers et al., 2024). Altogether, this suggests that shNoct CAD cells have more available resources for combating oxidative stress such as increased total glutathione levels (Figure 2C) and a lower rate of glutathione depletion (Figure 3F). Therefore, the shNoct CAD cells show a general enhancement in oxidative stress defense.

While we were able to observe positive effects due to the loss of Nocturnin expression on neuronal survival *in vitro*, a limitation of using the CAD cell line is that it does not produce dopamine. While CAD cells produce tyrosine hydroxylase (TH) and have been shown to produce and accumulate L-DOPA, which are precursors to dopamine production, they lack the enzymatic capacity to make dopamine (Pasuit et al., 2004; Qi et al., 1997). We had hypothesized that loss of Nocturnin would be able to reduce oxidative stress and promote neuron survival. Therefore, we utilized CAD cells for their neuronal properties, robust expression of Nocturnin, and ease of use in culture. Due to these limitations in CAD cells, we wanted to further explore the effect of Nocturnin in a more disease-relevant context *in vivo*. We focused on dopaminergic neurons in PD given the connection between Nocturnin, NADPH metabolism, and PD pathology in the literature (Charbonnier-Beaupel et al., 2015; Heiman et al., 2014; Simunovic et al., 2008; Yang et al., 2025; Yang et al., 2008; Zhou et al., 2018). DA_SYN53_ mice overexpress mutant human alpha-synuclein leading to robust dopaminergic neurodegeneration (Chen et al., 2015; Figures 4C,D). We utilized this PD mouse model to study whether deletion of Nocturnin affects dopaminergic neuron survival *in vivo*. While we confirmed the overexpression of human alpha-synuclein in these mice, we also observed increased mouse alpha-synuclein levels in the Noct^−/−^ mice, while expression remained comparable to WT levels in the other genotypes. This may suggest an indirect relationship between Nocturnin and endogenous alpha-synuclein expression, particularly given that alpha-synuclein levels do not increase in the DASYN53/Noct^−/−^ mice. Notably, the DA_SYN53_; Noct^−/−^ mice showed a rescue of TH + neurons that corresponded with an increase in NeuN+ neurons (Figures 4C,D). We observed a greater reduction in TH + neuron number in our DA_SYN53_ mice compared to the initial study in which the mouse model was first reported (Chen et al., 2015), potentially due to technical differences in fluorescence vs. colorimetric immunostaining, providing increased sensitivity and precision during stereology. Additionally, immunofluorescence staining allowed us to simultaneously quantify both TH + and NeuN+ populations within the same tissue areas analyzed by stereology, which provided greater confidence in the loss of TH staining. However, a limitation of this model is the DA_SYN53_ mice do not exhibit significant motor behavioral phenotypes (Chen et al., 2015), which is consistent with other PD mouse models (Beal, 2010). Because we did not explore the effects of the circadian regulation of Nocturnin expression and the presence of non-motor symptoms presented by NDD patients, future experiments will focus on time-of-day and additional behavioral phenotypes like sleep and eating patterns in future analyses of Nocturnin in alternative neurodegenerative disease models.

While oxidative stress markers have not been well defined in the DA_SYN53_ mice, impaired mitophagy suggests that oxidative stress is prevalent in this model (Chen et al., 2015). Alpha-synuclein dysfunction contributes to mitochondrial dysfunction, impaired dopamine metabolism, and increased oxidative stress in PD (Calabresi et al., 2023). Another study demonstrated that knocking out DJ-1, a regulator of oxidative stress defenses and an associated loss-of-function mutation linked to Parkinson’s disease, exacerbated alpha-synuclein-mediated dysfunction in the DA_SYN53_ mouse model (Burbulla et al., 2017). The effects of alpha-synuclein dysfunction are further illustrated in other PD models, including both genetic and pharmacological inducers, such as *pink1* knockout mice and MPTP-treated mice (Dauer et al., 2002; Vila et al., 2000). In this study, we show the loss of Nocturnin partially rescues neuronal loss in DA_SYN53_ mice, suggesting that oxidative stress is a key component of dopaminergic neurodegeneration following alpha synuclein overexpression and that knockout of Nocturnin aids in mitigating this oxidative stress-mediated neuronal cell loss. This corresponds with a previous study using a *pink1^−/−^* Drosophila model which demonstrated that Nocturnin knockdown rescued aberrant mitochondrial fusion, a process that can prevent functional mitophagy in PD (Yang et al., 2008). Given the established intersection between mitochondrial dysfunction, either driven by genetic mutations such as *pink1* or as a downstream consequence of disease progression, and oxidative stress, future studies should investigate whether Nocturnin influences mitochondrial dynamics or lysosomal integrity both *in vitro* and *in vivo*. Exploring these pathways could further clarify the subcellular role of Nocturnin in NDD contexts.

Overall, this study shows Nocturnin depletion significantly enhances neuronal viability, both *in vitro* and *in vivo*, highlighting its strong potential as a therapeutic target in a disease-relevant context. Due to the dichotomy that is present for molecules and proteins involved in redox homeostasis, it raises the question concerning the primary role of Nocturnin in neurons and the potential benefits of Nocturnin activity at physiological conditions. The answer may lie in the circadian behavior of Nocturnin. Nocturnin is ubiquitously expressed throughout the body and is known to have peak expression in the early evening and low expression by morning in mice (Wang et al., 2001). In addition to the regulation of daily gene expression patterns, the circadian clock is also known for its role in maintaining redox homeostasis. Studies reveal a reciprocal relationship between the circadian clock and redox factors, with disruptions in circadian rhythms contributing to neurodegeneration (Musiek et al., 2013; del Olmo et al., 2024; Rutter et al., 2001). For example, PD patients are known to have dysfunctional circadian rhythms and significant sleep disruptions (Titova and Chaudhuri, 2018; Videnovic et al., 2014). More specifically, NADPH is shown to help regulate sleep in *Drosophila* and disruption of the circadian clock was shown to sensitize dopaminergic neurons to oxidative stress mediated degeneration (Kempf et al., 2019; Majcin Dorcikova et al., 2023; Mariano et al., 2023). Therefore, we hypothesize that Nocturnin’s activity may be to act as a regulator of NADPH availability to aid in circadian regulation of redox homeostasis, which will need to be studied further. When circadian disruption occurs during disease progression, Nocturnin expression may persist constitutively or during impromptu periods during the day, causing ROS accumulation and decreased generation of antioxidants. Therefore, while this study focused on the overall loss of Nocturnin, a limitation is that we did not assess the specific contribution of its circadian regulation. An important future direction is to examine how the loss of Nocturnin’s circadian regulation, rather than its complete absence alone, contributes to the observed effects. Overall, regulation of NADPH levels by Nocturnin likely contributes to both redox homeostasis at physiological conditions, and pathophysiological cellular toxicity in NDDs like PD.

## Data Availability

The original contributions presented in the study are included in the article/Supplementary material, further inquiries can be directed to the corresponding author.
